# RETRACTION: Swimming Exercise Protects against Insulin Resistance Via Regulating Oxidative Stress through Nox4 and AKT Signaling in High‐Fat Diet‐Fed Mice

**DOI:** 10.1155/jdr/9762782

**Published:** 2025-12-12

RETRACTION: J. Qi, X. Luo, Z. Ma, et al. “Swimming Exercise Protects against Insulin Resistance via Regulating Oxidative Stress through Nox4 and AKT Signaling in High‐Fat Diet‐Fed Mice,” *Journal of Diabetes Research*, 2020, 2521590, https://doi.org/10.1155/2020/2521590

The above article, published online on 21 January 2020 in Wiley Online Library (http://wileyonlinelibrary.com), has been retracted by agreement between the journal Associate Editor, Birght Starling Emerald, and John Wiley & Sons Ltd. The retraction has been agreed due to concerns regarding the reliability of the data, specifically regarding the primers used for qPCRs and the antibodies used for immunoblotting to detect NOX4.

The authors did not provide a response or raw data for the above concerns, and following assessment by the editorial board, the article is retracted.

The authors did not respond to the retraction.

